# Metabolic Engineering of Microorganisms for the Production of Flavonoids

**DOI:** 10.3389/fbioe.2020.589069

**Published:** 2020-10-07

**Authors:** Huakang Sheng, Xinxiao Sun, Yajun Yan, Qipeng Yuan, Jia Wang, Xiaolin Shen

**Affiliations:** ^1^State Key Laboratory of Chemical Raesource Engineering, Beijing University of Chemical Technology, Beijing, China; ^2^College of Engineering, University of Georgia, Athens, GA, United States

**Keywords:** metabolic engineering, natural products, flavonoids, pathway optimization, microorganism

## Abstract

Flavonoids are a class of secondary metabolites found in plant and fungus. They have been widely used in food, pharmaceutical, and nutraceutical industries owing to their significant biological activities, such as antiaging, antioxidant, anti-inflammatory, and anticancer. However, the traditional approaches for the production of flavonoids including chemical synthesis and plant extraction involved hazardous materials and complicated processes and also suffered from low product titer and yield. Microbial synthesis of flavonoids from renewable biomass such as glucose and xylose has been considered as a sustainable and environmentally friendly method for large-scale production of flavonoids. Recently, construction of microbial cell factories for efficient biosynthesis of flavonoids has gained much attention. In this article, we summarize the recent advances in microbial synthesis of flavonoids including flavanones, flavones, isoflavones, flavonols, flavanols, and anthocyanins. We put emphasis on developing pathway construction and optimization strategies to biosynthesize flavonoids and to improve their titer and yield. Then, we discuss the current challenges and future perspectives on successful strain development for large-scale production of flavonoids in an industrial level.

## Introduction

**FIGURE 1:**
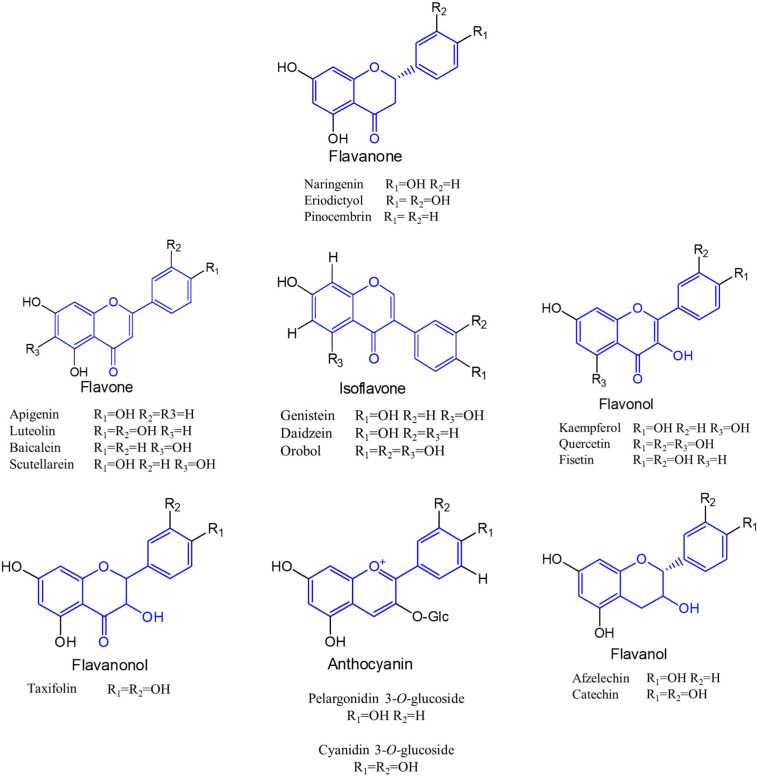
Molecular structures of different subclasses of flavonoids. The backbone of each subclasses is colored by blue.

Flavonoids are a special class of naturally occurring secondary metabolites that are synthesized by plant (Pandey et al., 2016) and fungus (Correa et al., 2011). Chemically, all flavonoids possess general structure of a 15-carbon skeleton with two phenyl rings (rings A and B) connected by a heterocyclic ring (ring C). In flavonoids biosynthetic pathways, ring A is formed from malonyl-CoA, and ring B is generated from 4-coumaroyl-CoA which is synthesized from phenylalanine via the shikimate pathway (Averesch and Krömer, 2018). Based on the chemical structures, flavonoids can be divided into several different subcategories, such as chalcones, flavanones, flavones, isoflavones, flavonols, flavanonols, flavanols, and anthocyanins (Tohge et al., 2017) (Figure 1).

Flavonoids have many important functions in plant, such as producing pigmentation for flower coloration (Nakayama et al., 2000), symbiotic nitrogen fixation (Fox et al., 2001), and UV protection (Kootstra, 1994). In addition, flavonoids demonstrate significant physiological and biochemical effects on mammals because of their unique chemical structures. Most flavonoids exhibit great antioxidant (Naderi et al., 2003), anti-inflammatory (Kim et al., 2004), antibacterial (Cushnie and Lamb, 2011), and anticancer (Ravishankar et al., 2013) activities; thus, they have been widely used in food, nutraceutical, and pharmaceutical industries (Kay et al., 2012; Georgiev et al., 2014; Hajimehdipoor et al., 2014). For example, apigenin can be used to treat high-fat and high-fructose diet–induced metabolic syndrome by reducing leptin levels and increasing adiponectin levels (Zhang et al., 2018). Epigallocatechin gallate and naringenin are able to maintain blood lipid and fat balance (Zhang et al., 2018). Quercetin, genistein, and naringenin have significant effect on reducing the expression levels of proinflammatory cytokines tumor necrosis factor α and interleukin 6 (Zhang et al., 2018). Owing to their significant bioactivities, the global market of flavonoids is rapidly growing and is expected to reach $1.05 billion in 2021 (Shah et al., 2019).

Currently, production of flavonoids predominantly relies on plant extraction, which is limited by difficulties in access and supply of the raw materials, low abundance and availability in plants, and low product yield (Jiang et al., 2005; Pyne et al., 2019). Chemical methods for production of flavonoids are also not preferred because of the complicated processes, strict reaction conditions, and poor selectivity (Pyne et al., 2019). Alternatively, introduction of the native biosynthetic pathways of flavonoids from plants into the microbial hosts through synthetic biology and metabolic engineering strategies represents a feasible approach for the production of flavonoids. In 2003, microbial synthesis of flavonoids was reported for the first time; since then, various flavonoids biosynthetic pathways have been successfully constructed in microorganisms (Hwang et al., 2003). Recently, construction of microbial cell factories for efficient production of flavonoids has made great progress (Pei et al., 2020; Wang et al., 2020; Yang et al., 2020). In this article, we recapitulate the recent and notable achievements on metabolic engineering and synthetic biology toward synthesis of flavonoids with relevant example cases (Table 1). Effects to construct flavonoids artificial biosynthetic pathways in microbes and to optimize products titer, yield, and productivity are presented.

**TABLE 1:** Metabolic engineering of flavonoids production in microorganisms.

**Subclass**	**Host strain**	**Substrate**	**Product**	**Titer**	**References**
Flavanone	*C. glutamicum*	*p*-Coumaric acid	Naringenin	35 mg/L	Kallscheuer et al. (2016)
	*C. glutamicum*	Caffeic acid	Eriodictyol	37 mg/L	Kallscheuer et al. (2016)
	*S. cerevisiae*	*p*-Coumaric acid	Naringenin	648.63 mg/L	Gao et al. (2019)
	*E. coli*	Glucose	Naringenin	84mg/L	Santos et al. (2011)
	*S. cerevisiae*	Glucose	Naringenin	112.90 mg/L	Koopman et al. (2012)
	*E. coli*	*p*-Coumaric acid	Naringenin	155 mg/L	Leonard et al. (2008)
	*E. coli*	Glucose	Pinocembrin	429 mg/L	Leonard et al. (2007)
	*E. coli*	Tyrosine	Naringenin	421.6 mg/L	Wu et al. (2015)
	*S. cerevisiae*	Glucose	Naringenin	90 mg/L	Lyu et al. (2017)
	*E. coli*	Tyrosine	Naringenin	191.9 mg/L	Zhou et al. (2019)
	*E. coli*	Naringenin	Eriodictyol	62.7 mg/L	Jones et al. (2016a)
	*S. cerevisiae*	Naringenin	Eriodictyol	200 mg/L	Amor et al. (2010)
	*E. coli*	Caffeic acid	Eriodictyol	114 mg/L	Fowler et al. (2009)
	*E. coli*	Glucose	Eriodictyol	5.7 mg/L	Zhu et al. (2014)
	*E. coli*	L-tyrosine	Eriodictyol	107 mg/L	Zhu et al. (2014)
	*S. cerevisiae*	Glucose	Eriodictyol	152 mg/L	Eichenberger et al. (2018)
Flavone	*E. coli*	*p*-Coumaric acid	Apigenin	415 μg/L	Leonard et al. (2006)
	*E. coli*	*p*-Coumaric acid	Apigenin	30 mg/L	Lee et al. (2015)
	*E. coli*	Glucose	Genkwanin	41 mg/L	Lee et al. (2015)
	*S. albus*	Glucose	Luteolin	0.09 mg/L	Marín et al. (2017)
	*E. coli*	L-phenylalanine	Baicalein	23.6 mg/L	Li et al. (2019)
	*E. coli*	L-tyrosine	Scutellarein	106.5 mg/L	Li et al. (2019)
	*E. coli*	*p*-Coumaric acid	Apigetrin (Apigenin-7-*O*-β-D-glucoside)	16.6 mg/L	Thuan et al. (2018)
	*S. cerevisiae*	Glucose	Scutellarin	108 mg/L	Liu et al. (2018)
	*S. cerevisiae*	Glucose	Apigenin-7-*O*-glucuronide	185 mg/L	Liu et al. (2018)
	*E. coli*	Apigenin	Isovitexin	3,772 mg/L	Pei et al. (2020)
	*E. coli*	Luteolin	Isoorientin	3,829 mg/L	Pei et al. (2020)
Isoflavone	*E. coli–S. cerevisiae* coculture	Tyrosine	Genistein	6 mg/L	Katsuyama et al. (2007)
	*E. coli–S. cerevisiae* coculture	Tyrosine	Genistein	100 mg/L	Horinouchi (2009)
	*S. cerevisiae*	Phenylalanine	Genistein	0.1 mg/L	Trantas et al. (2009)
	*E. coli*	Naringenin	Genistein	10 mg/g DCW	Leonard and Koffas (2007)
	*E. coli*	Liquiritigenin	Daidzein	18 mg/g DCW	Leonard and Koffas (2007)
	*E. coli*	Naringenin	Genistein	16 mg/L	Kim et al. (2009)
	*P. pastoris*	Genistein	Orobol	3.5 mg/L	Ding et al. (2015)
	*P. pastoris*	Genistein	Orobol	23 mg/L	Wang et al. (2016)
	*P. pastoris*	Daidzein	8-Hydroxydaidzein	0.58 mg/L	Chang et al. (2013)
	*P. pastoris*	Daidzein	3′-Hydroxydaidzein	0.23 mg/L	Chang et al. (2013)
	*P. pastoris*	Daidzein	6-Hydroxydaidzein	9.1 mg/L	Chang et al. (2013)
	*B. subtilis*	Genistein	Orobol	286 mg/L	Abari and Tayebi (2019)
	*E. coli*	Genistein	4′-*O*-methyl-genistein	48.61 mg/L	Koirala et al. (2019)
		Daidzein	4′-*O*-methyl-daidzein	102.88 mg/L	
	*E. coli*	Genistein	5,7,4′-Trihydroxy-3′-methoxyisoflavone	Detected	Chiang et al. (2017)
	*E. coli*	Genistein	5,7,3′-Trihydroxy-4′-methoxyisoflavone	Detected	Chiang et al. (2017)
	*E. coli*	Genistein	Genistein 7-*O*-glucoside	16.4 mg/L	He et al. (2008)
	*E. coli*	Genistein	Biochanin A 7-*O*-glucoside	11.7 mg/L	He et al. (2008)
Flavonol	*S. cerevisiae*	Glucose	Kaempferol	86 mg/L	Lyu et al. (2019)
	*S. cerevisiae*	Naringenin	Quercetin	0.38 mg/L	Trantas et al. (2009)
	*S. albus*	Glucose	Quercetin	0.1 mg/L	Marín et al. (2018)
	*E. coli*	Tyrosine	Fisetin	0.3 mg/L	Stahlhut et al. (2015)
	*S. cerevisiae*	Glucose	Kaempferol	26.6 mg/L	Rodriguez et al. (2017)
	*S. cerevisiae*	Glucose	Quercetin	20.4 mg/L	Rodriguez et al. (2017)
	*S. cerevisiae*	Glucose	Fisetin	2.3 mg/L	Rodriguez et al. (2017)
Flavanonol	*Y. lipolytica*	Glucose	Taxifolin	48.1 mg/L	Lv et al. (2019a)
	*Y. lipolytica*	Glucose	Taxifolin	110.5 mg/L	Lv et al. (2019b)
	*E. coli*	Taxifolin	Silybin and isosilybin	2.58 g/L	Lv et al. (2019c)
	*S. cerevisiae*	Glucose	Silybin	104.9 mg/L	Yang et al. (2020)
	*S. cerevisiae*	Glucose	Isosilybin	192.3 mg/L	Yang et al. (2020)
Flavanol	*E. coli*	Glucose	Afzelechin	26.1 mg/L	Jones et al. (2017)
	*E. coli*	Eriodictyol	Catechin	910.1 mg/L	Zhao et al. (2015)
	*E. coli*	Afzelechin	Catechin	34.7 mg/L	Jones et al. (2016a)
Anthocyanin	*E. coli*	Naringenin	Pelargonidin 3-*O*-glucoside	5.6 μg/L	Yan et al. (2005)
	*E. coli*	Eriodictyol	Cyanidin 3-*O*-glucoside	6.0 μg/L	Yan et al. (2005)
	*E. coli*	Naringenin	Pelargonidin 3-*O*-glucoside	78.9 mg/L	Yan et al. (2008)
	*E. coli*	Eriodictyol	Cyanidin 3-*O*-glucoside	70.7 mg/L	Yan et al. (2008)
	*E. coli*	Glucose	Pelargonidin 3-*O*-glucoside	9.5 mg/L	Jones et al. (2017)
	*E. coli*	Catechin	Cyanidin 3-*O*-glucoside	439 mg/L	Shrestha et al. (2019)
	*E. coli*	Catechin	Peonidin 3-*O*-glucoside	56 mg/L	Cress et al. (2017)
	*E. coli*	Glucose + (+)-catechin + tyrosine	pyranocyanidin-3-O-glucoside-phenol	19.5 mg/L	Akdemir et al. (2019)
	*E. coli*	Glucose + (+)-catechin	pyranocyanidin-3-O-glucoside-catechol	13 mg/L	Akdemir et al. (2019)
	*S. cerevisiae*	Glucose	Pelargonidin 3-*O*-glucoside	0.85 mg/L	Eichenberger et al. (2018)
	*S. cerevisiae*	Glucose	Cyanidin 3-*O*-glucoside	1.55 mg/L	Eichenberger et al. (2018)
	*S. cerevisiae*	Glucose	Delphinidin 3-*O*-glucoside	1.86 mg/L	Eichenberger et al. (2018)
	*S. cerevisiae*	Glucose	Pelargonidin	0.01 μmol/g CDW	Levisson et al. (2018)
	*S. cerevisiae*	Glucose	Pelargonidin 3-*O*-glucoside	0.001 μmol/g CDW	Levisson et al. (2018)
	*C. glutamicum*	Catechin	Cyanidin 3-*O*-glucoside	40 mg/L	Zha et al. (2018)
	*L. lactis*	Tea	Anthocyanins	1.5 mg/L	Solopova et al. (2019)

## Metabolic Engineering of Microorganisms for Flavonoids Production

**FIGURE 2:**
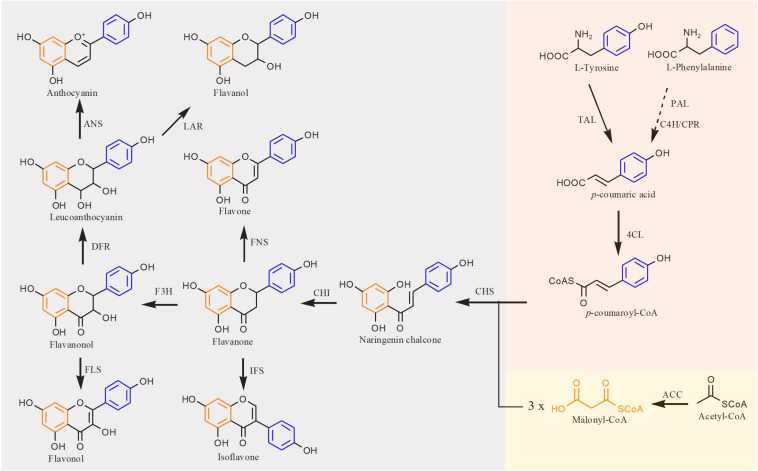
Biosynthetic pathways of flavonoid in plants. TAL, tyrosine ammonia lyase; PAL, phenylalanine ammonia lyase; C4H, cinnamate 4-hydroxylase; CPR, cytochrome P450 reductase; 4CL, 4-coumaroyl-CoA ligase; ACC, acetyl-CoA carboxylase; CHS, chalcone synthase; CHI, chalcone isomerase; FNS, flavone synthase; IFS, isoflavone synthase; F3H, flavanone 3β-hydroxylase; FLS, flavonol synthase; DFR, dihydroflavonol reductase; LAR, leucoanthocyanidin reductase; ANS, anthocyanidin synthase. Solid lines indicate a single step, and dotted lines indicate multiple steps.

The flavonoid biosynthetic pathway in plants has been clearly illustrated that extends from the shikimate pathway (Falcone Ferreyra et al., 2012) (Figure 2). In this pathway, the native precursors phenylalanine and tyrosine are converted to *p*-coumaroyl-CoA. Then, one molecule of *p*-coumaroyl-CoA and three molecules of malonyl CoA are converted to naringenin chalcone via Claisen cyclization under the action of chalcone synthase (CHS). Sequentially, the naringenin chalcone is converted to naringenin automatically or catalyzed by the chalcone isomerase (CHI). The produced naringenin in this pathway serves as an important intermediate to synthesize other classes of flavonoids. For example, flavones are formed by expression of flavone synthase (FNS). Isoflavonoids are generated by introduction of isoflavone synthase (IFS). Coexpression of flavonoid 3-hydroxylase (F3H) and flavonol synthase (FLS) results in flavonols, whereas flavanols can be produced by coexpressing F3H, dihydroflavonol 4-reductase (DFR), and leucoanthocyanidin reductase (LAR), and further introduction of anthocyanidin synthase (ANS) into this pathway can generate anthocyanins. In recent years, with the development of metabolic engineering and synthetic biology, more and more flavonoids have been achieved biosynthesis (Shah et al., 2019; Nabavi et al., 2020).

### Flavanones

Flavanones, including naringenin, pinocembrin and eriodictyol, are the first flavonoid products in flavonoids biosynthetic pathway. They are generally present in citrus fruits, such as grapefruit (Peterson et al., 2006), oranges (Khan et al., 2010), and lemons (Peterson et al., 2006) and have anti-inflammatory (Bano et al., 2013), antioxidant (Miranda et al., 2000), anticancer (Ketabforoosh et al., 2014), and other important pharmacological activities. The basic skeleton of flavanones is 2-phenylchromogen, which is characterized by the saturation of the C2–C3 double bond. The structure of flavanones is highly active that can be modified by hydroxylation (Britsch and Grisebach, 1986), methylation (Koirala et al., 2016), and glycosylation (Di Majo et al., 2005). Biosynthesis of naringenin from the precursor *p*-coumaric acid has been achieved in *Corynebacterium glutamicum* by overexpression of CHS and CHI from *Petunia hybrida*. By disrupting the competing pathways, 35 mg/L naringenin were produced in the engineered host strains (Kallscheuer et al., 2016). It was believed that the low titers were limited by the low enzyme activity of CHS. Alternatively, Gao et al. (2019) introduced the *SmCHS2* from *Silybum marianum* into *Saccharomyces cerevisiae* and obtained 648.63 mg/L naringenin by exogenously feeding *p*-coumaric acid.

*De novo* biosynthesis of flavanones from simple carbon sources has also been achieved. Santos et al. introduced the genes *RgTAL* (encoding tyrosine ammonia lyase) from *Rhodotorula glutinosa*, *Pc4CL* (encoding 4-coumaric acid-CoA ligase) from *Petroselinum crispum*, *PhCHS* from *P. hybrida*, and *MsCHI* from *Medicago sativa* into a tyrosine-overproducing *S. cerevisiae* strain, resulting in 29 mg/L naringenin using glucose as the substrate (Santos et al., 2011). In another example, the naringenin biosynthetic pathway in *Arabidopsis thaliana* containing phenylalanine ammonia lyase (PAL), cinnamate 4-hydroxylase (C4H), cytochrome P450 reductase (CPR), *4CL*, *CHS*, and CHI was transferred into the *S. cerevisiae*. The original recombinant host strain only generated 1.4 mg/L naringenin from glucose. To further improve the titer, the feedback inhibition effect was eliminated by deletion of *ARO3* and introduction of the *ARO4*^*G*226*S*^ mutant. As a result, the naringenin production increased to 2.8 mg/L. Furthermore, disruption of competing pathway and enhancement of the precursor *p*-coumaric acid supply enabled the host strain to produce 54.5 mg/L naringenin in shake flask experiments (Koopman et al., 2012). The insufficient precursor supply is still the dominant rate-limiting factors for high-level production of naringenin.

Malonyl-CoA not only serves as an important precursor for the production of flavanones but also is an essential intermediate for the synthesis of fatty acids to support cell growth in microorganisms (Takamura and Nomura, 1988). Thus, there exist a trade-off relationship between the flavanone biosynthesis and the cell growth. In order to balance the production phase and growth phase, Leonard et al. (2008) constructed a malonate assimilation pathway in a recombinant naringenin-producing *Escherichia coli* strain by overexpression of the malonate synthase (MatB) and malonate carrier protein (MatC) isolated from *Rhizobium trifolii*. Introduction of malonate assimilation pathway enables engineered *E. coli* strain to synthesize malonyl-CoA by exogenously feeding malonate. Finally, the recombinant *E. coli* strain produced 155 mg/L of naringenin, indicating a 2.7-fold improvement in titer compared with the parent strain without malonate assimilation pathway overexpression (Leonard et al., 2008). Cerulenin is a fatty acid synthase inhibitor for inhibiting the expression levels of *fabB* and *fabF*. According to the report, addition of 200 μM of cerulenin could increase the concentration of malonyl-CoA from 2 pmol/mg cell dry weight (CDW) to 105 pmol/mg CDW in 1.5 h (van Summeren-Wesenhagen and Marienhagen, 2015). By exogenously supplementing cerulenin into the cultures, the naringenin titer enhanced from 29 mg/L to 84 mg/L in engineered host strains (Santos et al., 2011). Although those strategies can effectively increase the level of malonyl-CoA in engineered host strain, thereby to enhance the titer of flavanones, the high costs of malonate and cerulenin limited their use in high-level production of flavanones. In microbes, malonyl-CoA is formed from acetyl-CoA catalyzed by acetyl-CoA carboxylase complex (ACC). Leonard et al. (2007) introduced *ACC* from *Pseudomonas luminescens* into *E. coli*; the titer of pinocembrin reached 196 mg/L, exhibiting a 5.8-fold increase in titer compared with that in control strains. Additionally, the availability of malonyl-CoA could also be improved by employing a clustered regularly interspaced short palindromic repeat interference (CRISPRi) system to inhibit the expression level of genes involved in essential metabolic pathways. For instance, Wu et al. (2015) utilized the CRISPRi system to down-regulate the expression level of genes *fabF*, *fumC*, *fabB*, *sucC*, and *adhE* in recombinant *E. coli* to redirect the carbon flux toward malonyl-CoA synthesis. The resultant strain generated 421.6 mg/L naringenin from tyrosine, which is the highest naringenin production reported so far (Wu et al., 2015). Recently, Lyu et al. (2017) tried to improve naringenin titer in *S. cerevisiae* by dividing the naringenin biosynthetic pathway into three modules: the first module containing TAL, 4CL, CHS, and CHI was applied for naringenin biosynthesis. The second module carrying acetyl-CoA synthase, ATP-citrate lyase, and ACC was used for malonyl-CoA accumulation, whereas the third module harboring the *ARO4*^*K*229*L*^ mutant was employed to produce more tyrosine. Engineering and integration of those three modules into the final strain resulted in 90 mg/L of naringenin from glucose (Lyu et al., 2017). In another example, an iterative high-throughput screening method was applied to fine-tune the expression level of naringenin biosynthetic pathway genes. A promoter library containing a series of constitutive promoters with different strength was constructed. Those promoters were randomly placed upstream of naringenin biosynthetic pathway genes. After several rounds of high-throughput screening, the best performed recombinant *E. coli* strain produced 191.9 mg/L naringenin, representing a two-fold increase in titer compared with that in a strain optimized using traditional modular optimization strategy (Zhou et al., 2019).

On the basis of the naringenin biosynthetic pathway, eriodictyol can be produced by introducing F3′H into this pathway. A 4-hydroxyphenylacetate 3-hydroxylase (EcHpaBC) was identified and characterized from *E. coli*. It is able to directly transform the naringenin into eriodictyol with 62.7 mg/L of eriodictyol produced (Jones et al., 2016a). In another example, a cytochrome P450 F3′H isolated from *Gerbera hybrida* was functionally expressed in *S. cerevisiae*, resulting in 200 mg/L eriodictyol from naringenin (Amor et al., 2010). In addition, by directly feeding caffeic acid rather than *p*-coumaric acid as the precursor, eriodictyol can also be biosynthesized via overexpressing 4CL, CHS, and CHI. Fowler et al. (2009) introduced 4CL, CHS, and CHI into *E. coli*; 114 mg/L eriodictyol was obtained by exogenously supplementing caffeic acid into the cultures. *De novo* biosynthesis of eriodictyol from simple carbon source in *E. coli* was also achieved by coexpressing TAL, 4CL, CHS, and CHI with the cytochrome P450 F3′H. In this study, a fusion protein (tF3′H-tCPR) was created by fusing a truncated F3′H with a truncated CPR to improve the solubility and enzyme activity of F3′H. Finally, only 5.7 mg/L of eriodictyol was obtained using glucose as the substrate (Zhu et al., 2014). The low catalytic efficiency of F3′H is still the rate-limiting factor in this pathway. It is believed that compared with yeasts, bacterial hosts are usually difficult to express cytochrome P450 enzymes because they are unable to perform posttranslational modifications and expression of membrane proteins. When transferring the *de novo* eriodictyol biosynthetic pathway into *S. cerevisiae*, the titer of eriodictyol improved to 152 mg/L from glucose (Eichenberger et al., 2018).

### Flavones

**FIGURE 3:**
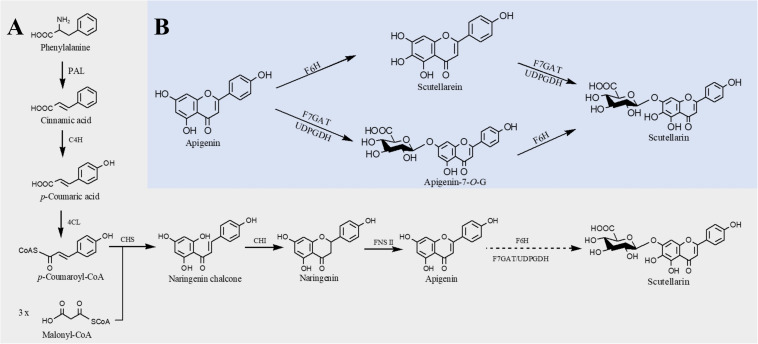
Microbial production of scutellarin in *E. coli*. **(A)** Biosynthesis of scutellarin from phenylalanine; **(B)** biosynthesis of scutellarin from apigenin. PAL, phenylalanine ammonia lyase; C4H, cinnamate 4-hydroxylase; 4CL, 4-coumaroyl-CoA ligase; CHS, chalcone synthase; CHI, chalcone isomerase; FNSII, flavone synthase II; F6H, flavone 6-hydroxylase; F7GAT, flavonoid-7-*O*-glucuronosyltransferase; UDPGDH, UDP-glucose dehydrogenase. Solid lines indicate a single step, and dotted lines indicate multiple steps.

Structurally, flavones correspond to a flavonoid subgroup that characterized by a 2-phenylchromogen-4-one backbone and have a double bond between C2 and C3. Flavones are commonly found in fruits and vegetables, such as carrots, parsley, mint, and red and chili peppers (Panche et al., 2016) and exhibit great health benefits such as anti-inflammatory (Huang and Ho, 2010), anticancer (Li-Weber, 2009), antioxidant (Škerget et al., 2005), hypoglycemic, and hypolipidemic activities (Sharma et al., 2008) and prevention of heart disease (Hirvonen et al., 2001). Apigenin, a common flavone, can be synthesized from naringenin catalyzed by FNS. The first case about biosynthesis of apigenin in microorganisms was reported in 2006. Leonard et al. (2006) introduced 4CL, CHS, CHI, and an FNS from *P. crispum* into *E. coli*; 415 μg/L of apigenin was produced from *p*-coumaric acid, and the conversion rate was only 2.9% (Leonard et al., 2006). Since then, continuous researches have been reported to improve the apigenin production. Lee et al. (2015) constructed an apigenin biosynthetic pathway in *E. coli* composed of 4CL from *Oryza sativa*, CHS from *Populus euramericana*, CHI from *Medicago truncatula* and FNS from *Parsley* in *E. coli*, 23 mg/L of apigenin was obtained from *p*-coumaric acid. Then the titer was further increased to 30 mg/L by enhancing the expression levels of 4CL and CHS (Lee et al., 2015). Hydroxylation of apigenin at the 3′ position results in luteolin. This reaction usually catalyzed by F3′H. Recently, Marin et al. constructed a *de novo* luteolin biosynthetic pathway in *Streptomyces albus* by employing the F3′H from *A. thaliana* and generated 0.09 mg/L of luteolin (Marín et al., 2017). Very recently, baicalein (5,6,7-trihydroxyflavone) and scutellarein (4′,5,6,7-tetrahydroxyflavone) have also been synthesized in *E. coli*. Overexpression of PAL from *Rhodotorula toruloides*, 4CL and FNS from *P. crispum*, CHS from *P. hybrida*, CHI from *M. sativa*, F6′H (encoding flavonoid 6′-hydroxylase) from *Scutellaria baicalensis*, and CPR from *A. thaliana*, baicalein, and scutellarein can be produced from tyrosine and phenylalanine, respectively (Figure 3). To remove the rate-limiting step, a hydrophilic modification 2B1 was incorporated with an N-terminal truncated F6′H to increase its solubility in *E. coli*, resulting in 8.5 mg/L baicalein and 47.1 mg/L scutellarein in shake flask experiments. Further enhancement of malonyl-CoA availability greatly improved the titers to 23.6 mg/L baicalein and 106.5 mg/L scutellarein (Li et al., 2019).

Except for hydroxylation, other modifications such as methylation and glycosylation also contribute to expand the structural diversity of flavones. For example, by introducing a methyl group at C7 site of apigenin, one compound known as genkwanin is obtained. Compared with apigenin, genkwanin exhibits many novel properties including antibacterial and radical scavenging activities. Lee et al. (2015) introduced apigenin 7-*O*-methyltransferase (POMT7) from *Populus deltoides* into an apigenin overproducing *E. coli* strain to construct a *de novo* biosynthetic pathway for genkwanin production; the titer of genkwanin reached 41 mg/L by using glucose as the carbon source. By introducing glucosyl group into the chemical structure of apigenin at the C7 site, the product apigenin-7-*O*-β-D-glucoside appears several additional bioactivities, such as antiproliferative and higher antioxidant activities. Tuan et al. achieved apigenin-7-*O*-β-D-glucoside production from *p*-coumaric acid in *E. coli* by employing a glycosyltransferase (PaGT3) from *Phytolacca americana*. To eliminate by-products formation, a coculture strategy was used by separating the entire pathway into two strains. The first strain contains 4CL from *Nicotiana tabacum*, CHS from *P. hybrida*, CHI from *M. sativa*, and FNS from *Parsley* to generate apigenin from *p*-coumaric acid, whereas the second strain carries *PaGT3* and an UDP-glucose overproduction system to convert apigenin into apigenin-7-*O*-β-D-glucoside. By optimization of initial inoculum ratio of the two strains, the titer of apigenin-7-*O*-β-D-glucoside reached 16.6 mg/L, which was 2.5 times higher than monoculture producing strain (Thuan et al., 2018). Breviscapine is a crude extract of flavonoids isolated from *Erigeron breviscapus*. It has been used for treatment of cardiovascular and cerebrovascular diseases for a long time (Zhong et al., 2005). The main active components in breviscapine are scutellarin and apigenin-7-*O*-glucuronide. Liu et al. (2018) identified two key enzymes involved in breviscapine biosynthetic pathway in *E. breviscapus*: one is flavonoid 7-*O*-glucuronosyltransferase, and the other is flavone-6-hydroxylase. Introduction of those two enzymes into an apigenin overproducing *S. cerevisiae* strain resulted in 108 mg/L of scutellarin and 185 mg/L of apigenin-7-*O*-glucuronide using glucose as the carbon source (Liu et al., 2018). Very recently, a *C*-glycosyltransferase (Gt6CGT) from *Gentiana triflora* was identified and characterized. It exhibits significant enzyme activities for conversion of apigenin and luteolin into isovitexin and isoorientin, respectively. This enzyme was coupled with a sucrose synthase (*GmSUS*) from *Glycine max* to regenerate the UDP-glucose. After optimizing coupled reaction conditions, the production of isovitexin and isoorientin reached 3,772 and 3,829 mg/L with molar conversion rate of 97.1 and 94.7%, respectively (Pei et al., 2020).

### Isoflavones

Isoflavones differ from flavones by the position of the phenyl group at the C3 site, which is at the C2 site in flavones. They represent a class of phytoestrogens produced by the leguminous plants (Kaufman et al., 1997; Chon, 2013) such as soybean, green bean, alfalfa sprout, and cowpea. Isoflavones act as estrogens on human body and can be used to treat various hormonal disorders such as prostate cancer (Kumar et al., 2004), breast cancer (Dong and Qin, 2011), osteoporosis (Taku et al., 2011), and cardiovascular diseases (Watanabe et al., 2002). Biosynthesis of isoflavones can be achieved from flavanones catalyzed by IFS, a membrane-bound cytochrome P450 monooxygenase. Utilization of naringenin and liquiritigenin as the substrates for IFS, resulting in the generation of genistein and daidzein, respectively. Katsuyama et al. (2007) introduced CHS, CHI, and an IFS isolated from *Glycyrrhiza echinata* into the yeast strain *S. cerevisiae*; 0.34 mg/L genistein was produced by feeding *N*-acetylcysteamine-attached *p*-coumaric acid as the precursor. To achieve biosynthesis of genistein from tyrosine, a naringenin overproducing *E. coli* strain, was cocultured with a recombinant *S. cerevisiae* carrying the *IFS* gene, which resulted in 6 mg/L genistein from tyrosine (Katsuyama et al., 2007). The titer was further enhanced to 100 mg/L by optimization of coculture conditions (Horinouchi, 2009). To avoid metabolite transport limitations through the cell walls of two different strains, it is beneficial to assemble entire pathway into a single strain. Trantas et al. (2009) reconstituted the entire genistein biosynthetic pathway in *S. cerevisiae* by coexpression of seven genes encoding PAL, CPR, C4H, 4CL, CHS, CHI, and IFS. The best engineered strain synthesized 0.1 mg/L genistein when the cultures were fed with phenylalanine as the precursor (Trantas et al., 2009). However, expression of IFS in bacteria is very difficult because of the absence of the electron transfer system. In order to functionally express IFS in *E. coli*, a redox partner CPR from *Catharanthus roseus* was fused to IFS from *G. max* by a glycine–serine–threonine linker sequence, which enabled it to efficiently transfer electron from NAD(P)H to substrate. Then, the *N*-terminus of this fusion protein was truncated and appended to a tailor-made membrane recognition signal. Expression of this construct in *E. coli* enabled efficient production of 10 and 18 mg/g dry cell weight of genistein and daidzein from naringenin and liquiritigenin, respectively (Leonard and Koffas, 2007). In another study, an IFS from red clover was truncated by removing the first 21 amino acids on the *N*-terminus, and then it was fused to a CPR from rice. The recombinant *E. coli* expressing this fusion protein can produce 16 mg/L genistein from naringenin (Kim et al., 2009).

The chemical structures of isoflavones also can be conjugated with hydroxyl (Tahara et al., 1991), methyl (Edwards and Dixon, 1991), and glycosyl (Ismail and Hayes, 2005) groups by hydroxylases, methyltransferases and glucosyltransferase. Overexpression of a P450 hydroxylase (CYP57B3) from *Aspergillus oryzae* and a CPR from *S. cerevisiae* in *Pichia pastoris* led to 3.5 mg/L of orobol (3′-hydroxygenistein) in a fed-batch culture fed with genistein as the precursor (Ding et al., 2015). Wang et al. further increased the titer of orobol to 23 mg/L by construction of a *P. pastoris* mutant strain via periodic hydrogen peroxide treatment (Wang et al., 2016). The CYP57B3 also has been used to hydroxylate daidzein in a recombinant *P. pastoris* strain. Interestingly, when using daidzein as the substrate, three orthohydroxylated daidzein derivatives were obtained, including 8-hydroxydaidzein (8-OHDe), 3′-hydroxydaidzein (3′-OHDe), and 6-hydroxydaidzein (6-OHDe), with a conversion rate of 2.4, 0.9, and 36.3%, respectively (Chang et al., 2013). In order to improve the catalytic efficiency of hydroxylase and avoid by-products formation, a tyrosinase from *Bacillus megaterium* was functionally displayed on *Bacillus subtilis* spores using CotE as an anchor protein (Hosseini-Abari et al., 2016). This spore displayed tyrosinase is able to efficiently hydroxylate 1 mM genistein into 1 mM orobol with a 100% conversion rate in 90 min (Abari and Tayebi, 2019). The diversity of isoflavones also can be expanded by methylation reactions. Koirala et al. (2019) constructed a recombinant *E. coli* strain for methylation of isoflavones by overexpressing native *EcSAM* encoding *S*-adenosy-L-methionine (SAM) synthase and *SaOMT2* encoding an *O*-methyltransferase from *Streptomyces avermitilis*. When using genistein and daidzein as the substrates, 4′-*O*-methyl-genistein and 4′-*O*-methyl-daidzein were obtained. After optimization of substrate concentrations, incubation time, and culture conditions, 46.81 mg/L of 4′-*O*-methyl-genistein and 102.88 mg/L of 4′-*O*-methyl-daidzein were generated (Koirala et al., 2019). In another example, genistein was converted into 5,7,3′-*O*-trihydroxy-4′-methoxyisoflavone and 5,7,4′-trihydroxy-3′-methoxyisoflavone by two sequential bioreactions including 3′-hydroxylation catalyzed by one recombinant *E. coli* strain containing tyrosinase from *B. megaterium* and then methylation catalyzed by another recombinant *E. coli* strain expressing *O*-methyltransferase from *Streptomyces peucetius* (Chiang et al., 2017).

By introduction of glucosyltransferases, the isoflavone glycosides can be produced. Heterologous overexpression of an UDP-glucosyltransferase UGT71G1 from *M. truncatula* enabled the recombinant *E. coli* strain to synthesize 16.4 mg/L genistein 7-*O*-glucoside and 11.7 mg/L biochanin A 7-*O*-glucoside from genistein and biochanin A in 500 mL TB culture medium (He et al., 2008).

### Flavonols and Flavanols

Flavonols can be considered as the derivatives of flavones; their structural difference is on the presence of a 3-hydroxy group on flavonols, which is lacking on flavones. Flavonols are commonly found in tea, red wines, fruits, and vegetables (Panche et al., 2016). Flavonols have a wide range of applications in pharmaceutical industries. For example, quercetin has been proven to be effectively inhibit the metastasis of melanoma cells (Caltagirone et al., 2000). Fisetin has the potential to treat neurodegenerative diseases (Fazel Nabavi et al., 2016) such as Alzheimer disease and Huntington disease. Biosynthesis of flavonols can be achieved by coexpressing F3H and FLS with flavanones as the precursor. Lyu et al. (2019) introduced F3H and FLS into a naringenin-producing *S. cerevisiae* strain Y-22, which rendered the recombinant strain the ability to generate kaempferol. Further optimization strategies including screening of efficient pathway enzymes, disruption of competing pathways, enhancement of the precursors PEP and E4P supply, and mitochondrial engineering of F3H and FLS significantly improved the kaempferol titer to 86 mg/L (Lyu et al., 2019). Quercetin is a 3′-hydroxylated derivative of kaempferol. Biosynthesis of quercetin was achieved by extending kaempferol biosynthetic pathway via overexpressing F3′H in *S. cerevisiae.* When naringenin was added into the cultures, the best engineered host strain produced 0.38 mg/L of quercetin during 70-h cultivation (Trantas et al., 2009). Recently, *de novo* biosynthesis of quercetin in actinomycetes was reported for the first time. In this study, an artificial pathway containing the TAL from *Rhodobacter capsulatus*, 4CL from *S. coelicolor*, CHS and CHI from *G. max*, naringenin 3-dioxygenase from *P. crispum*, and FLS and F3′H from *A. thaliana* was constructed and introduced into *S. albus* and *S. coelicolor*. The recombinant *S. albus* produced the higher quercetin titer of 0.1 mg/L (Marín et al., 2018). Fisetin has very similar chemical structure with quercetin; hydroxylation of fisetin at the C5 site results in quercetin. According to the structural similarity between fisetin and quercetin, a novel biosynthetic pathway for the production of fisetin was developed by recruiting liquiritigenin as the intermediate. Heterologous overexpression of TAL, 4CL, CHS, and chalcone reductase (CHR) from *Astragalus mongholicus* and CHI, F3H, FLS, flavonoid 3′-monooxygenase (FMO), and CPR in *E. coli* resulted in 0.3 mg/L of fisetin from tyrosine (Stahlhut et al., 2015). Recently, *de novo* biosynthesis of kaempferol, quercetin, and fisetin from simple carbon source in *S. cerevisiae* was reported. The best engineered host strain produced 26.6 mg/L of kaempferol, 20.4 mg/L of quercetin, and 2.3 mg/L of fisetin directly from glucose (Rodriguez et al., 2017).

**FIGURE 4:**
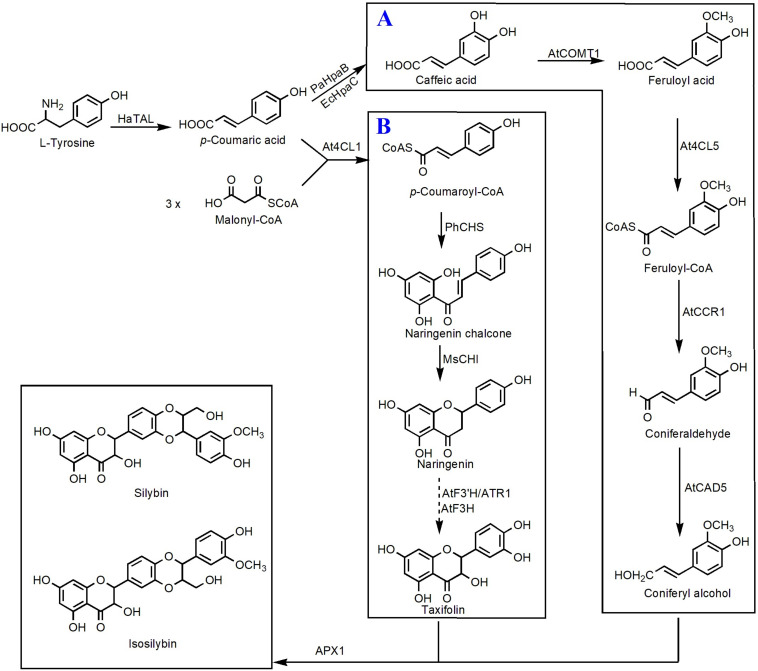
Microbial production of silybin and isosilybin in *S. cerevisiae*. **(A)** Biosynthesis of coniferyl alcohol from caffeic acid; **(B)** biosynthesis of taxifolin from *p*-coumaroyl-CoA. HaTAL, tyrosine ammonia lyase from *H. aurantiacus*; PaHpaB, 4-hydroxyphenylacetate 3-monooxygenase from *P. aeruginosa*; EcHpaC, flavin reductase from *E. coli*; AtCOMT1, *O*-methyltransferase 1 from *A. thaliana*; At4CL5, 4-coumarate-CoA 5 ligase from *A. thaliana*; AtCCR1, cinnamoyl-CoA reductase 1 from *A. thaliana*; AtCAD5, cinnamyl alcohol dehydrogenase 5 from *A. thaliana*; At4CL1, 4-coumarate-CoA 1 ligase from *A. thaliana*; PhCHS, chalcone synthase from *P. hybrida*; MsCHI, chalcone isomerase from *M. sativa*; AtF3′H, flavonoid 3′-monooxygenase from *A. thaliana*; ATR1, cytochrome P450 reductase 1 from *A. thaliana*; AtF3H, flavanone 3-hydroxylase from *A. thaliana*; APX1, ascorbate peroxidase 1 from *S. marianum*. Solid lines indicate a single step, and dotted lines indicate multiple steps.

Taxifolin, also known as dihydroquercetin, belongs to the flavanonols. Biosynthesis of taxifolin can be achieved by employment of eriodictyol as the direct precursor under the catalysis of F3H. Recently, Lv et al. (2019a) constructed a taxifolin biosynthetic pathway consisting TAL, 4CL, CHS, CHI, F3′H, CPR, and an F3H from *Solanum lycopersicum*. Integration of this pathway into the genome of *Yarrowia lipolytica* resulted in 48.1 mg/L of taxifolin (Lv et al., 2019a). Then, several metabolic engineering optimization strategies, such as improving precursor supply, enhancing copy number of the rate-limiting enzyme CHS, and screening efficient CPR, were utilized to increase the taxifolin titer. The final engineered strain generated 110.5 mg/L taxifolin using glucose as the carbon source (Lv et al., 2019b). Taxifolin can be used as the precursor to synthesize other important compounds. Very recently, Lv et al. (2019c) introduced a vanillyl alcohol oxidase (*PsVAO*) from *Penicillium simplicissimum* and an ascorbate peroxidase (*APX1*) from *milk thistle* into *E. coli* strain to build up an enzymatic cascade that is able to efficiently convert taxifolin and eugenol to silybin and isosilybin. Under the best culture conditions, a total amount of 2.58 g/L silybin and isosilybin was generated with a molar conversion rate of 76.7% in a 3-L fermenter (Lv et al., 2019c). On this basis, Yang et al. (2020) achieved biosynthesis of silybin and isosilybin from glucose in *S. cerevisiae* for the first time (Figure 4). In this study, the biosynthetic pathways for production of coniferyl alcohol and taxifolin were individually reconstructed in two different strains. Systematic optimization of those two pathways enabled the host strains to respectively synthesize 201.1 mg/L of coniferyl alcohol and 336.8 mg/L of taxifolin. Using APX1 as the catalyst, 104.9 mg/L of silybin and 192.3 mg/L of isosilybin were produced from coniferyl alcohol and taxifolin, with a yield of 62.5% (Yang et al., 2020).

Flavanols, also known as flavan-3-ols, are occurring abundantly in fruits such as apple, peach, and pear (Panche et al., 2016). It has been reported that quite a high level of flavanols was detected in cocoa (Neukam et al., 2007). An overwhelming body of research evidence demonstrated that regular flavanols intake can prevent hypertension and cardiovascular and cerebrovascular diseases (Schroeter et al., 2006). Biosynthesis of flavanols can be achieved by extending the flavanones biosynthetic pathway via introduction of F3H, DFR, and LAR. Afzelechin and catechin are the most important representatives of the class of flavanols. Afzelechin is a typical flavan-3-ol, which is synthesized by using naringenin as the precursor. Recently, *de novo* biosynthesis of afzelechin from glucose in microorganism was achieved for the first time. In this study, a polyculture system was developed by separating the entire biosynthetic pathway into three parts: glucose to *p*-coumaric acid, *p*-coumaric acid to naringenin, and naringenin to afzelechin. Those three parts were individually introduced into three different *E. coli* strains. The best engineered production system generated 26.1 mg/L afzelechin in shake flask experiments (Jones et al., 2017). In another example, F3H from *Camellia sinensis*, DFR from *Fragaria ananassa*, and LAR from *Desmodium uncinatum* were coexpressed in *E. coli*, thus constructing a recombinant *E. coli* strain that is able to synthesize catechin using eriodictyol as the precursor. Enhancement of the NADPH availability, optimization of pathway gene copy number, and employment of a series of scaffolding proteins enabled the production of 910.1 mg/L catechin from eriodictyol, with a conversion rate of 91% (Zhao et al., 2015).

Alternatively, according to the structural similarity between catechin and afzelechin, it is believed that biosynthesis of catechin can also be achieved by hydroxylation of afzelechin at the C3′ site. Jones et al. employed the non-P450 hydroxylase EcHpaBC to directly hydroxylate afzelechin with catechin titer of 34.7 mg/L (Jones et al., 2016a).

### Anthocyanins

Anthocyanins are a class of flavonoids containing polyhydroxy or polymethoxy derivatives of 2-phenylbenzophyryllium in their chemical structures. They are naturally occurring pigments widespread in virous flowers, vegetables, fruits, and cereal grains (Jackman et al., 1987; Wrolstad, 2004). Anthocyanins present black, blue, purple, or red in color, depending on the pH of the environment (Heredia et al., 1998; Torskangerpoll and Andersen, 2005). Anthocyanins possess great antioxidant (Miguel, 2011), anticancer (Bowen-Forbes et al., 2010), and antidiabetic (Mojica et al., 2017) activities, thus exhibiting great health-promoting potentials. The biosynthetic pathway of anthocyanins extended from the flavanols biosynthetic pathway by overexpression of ANS and glycosyltransferases. Yan et al. (2005) constructed a four-step biosynthetic pathway for the production of anthocyanin in *E. coli* by introduction of F3H, DFR, an ANS from *Malus domestica* and a 3-*O*-glucosyltransferase (PGT8) from *P. hybrida*. As a result, 5.6 μg/L pelargonidin 3-*O*-glucoside and 6.0 μg/L cyanidin 3-*O*-glucoside were produced by exogenously adding naringenin and eriodictyol into the cultures (Yan et al., 2005). Further enhancement of the UDP-glucose supply and construction of an ANS-PGT8 fusion protein greatly improved the titers to 78.9 mg/L pelargonidin 3−*O*−glucoside and 70.7 mg/L cyanidin 3−*O*−glucoside (Yan et al., 2008). *De novo* biosynthesis of pelargonidin 3-*O*-glucoside is very difficult to achieve because the biosynthetic process is too complicated and involves too many enzymatic steps. Recently, to overcome this issue, a polyculture system was constructed to improve the titer of pelargonidin 3-*O*-glucoside. In this system, the entire pelargonidin 3-*O*-glucoside biosynthetic pathway containing 15 enzymes and transcriptional factors was distributed into four independent *E. coli* strains, which allowed for the distribution of metabolic burden. When using glucose as the carbon source, the best performed polyculture system produced 9.5 mg/L pelargonidin 3-*O*-glucoside (Jones et al., 2017). Very recently, Shrestha et al. (2019) constructed cyanidin 3-*O*-glucoside biosynthetic pathway in *E. coli* by overexpression of ANS from *P. hybrida* and cyanidin 3-*O*-glucosyltransferase (At3GT) from *A. thaliana*. In order to balance the expression level of *ANS* and *At3GT*, a synthetic bio-brick vector system was developed to express ANS, *At3GT*, and UDP-glucose biosynthetic pathway genes under different promoters. The results showed that one engineered strain carrying *At3GT* and ANS driven by the P_*trc*_ promoter and UDP-glucose biosynthetic pathway genes under P_*T*__7_ promoter resulted in the highest cyanidin 3-*O*-glucoside titer of 439 mg/L in 36-h cultivation (Shrestha et al., 2019). In another example, a methylated anthocyanin, peonidin 3-*O*-glucoside, was produced in engineered *E. coli* by extra expression of an *O*-methyltransferase. Screening of efficient pathway enzymes and improving the SAM availability via a CRISPRi system enhanced the peonidin 3-*O*-glucoside titer to 56 mg/L (Cress et al., 2017). Very recently, coculture systems were developed in *E. coli* strains to produce pyranoanthocyanins. In this work, 4-vinylphenol and 4-vinylcatechol producers were separately cocultured with cyanidin-3-*O*-glucoside producer cells to generate pyranocyanidin-3-*O*-glucoside-phenol and pyranocyanidin-3-*O*-glucoside-catechol. After optimization of the coculture conditions, up to 19.5 mg/L of pyranocyanidin-3-*O*-glucoside-phenol and 13 mg/L of pyranocyanidin-3-*O*-glucoside-catechol were synthesized from glucose (Akdemir et al., 2019).

Actually, *E. coli* is not a preferred host to produce anthocyanins because of too many cytochrome P450 enzymes involved in the biosynthetic pathway. Thus, a lot of efforts have been made to construct anthocyanins biosynthetic pathway in *S. cerevisiae*. For instance, Eichenberger et al. (2018) first reported *de novo* biosynthesis of pelargonidin-3-*O*-glucoside, cyanidin-3-*O*-glucoside, and delphinidin-3-*O*-glucoside in engineered *S. cerevisiae* strains. By screening of efficient glucosyltransferase, 0.85 mg/L pelargonidin 3−*O*−glucoside, 1.55 mg/L cyanidin 3-*O*-glucoside, and 1.86 mg/L delphinidin-3-*O*-glucoside were produced in the best engineered strains (Eichenberger et al., 2018). In another example, Levisson et al. (2018) constructed a biosynthetic pathway for the production of pelargonidin 3-*O*-glucoside starting from glucose in *S. cerevisiae.* The host strain was engineered to prevent by-product formation and to improve the precursors supply, the resultant strain generated 0.01 μmol/g CDW pelargonidin and 0.001 μmol/g CDW pelargonidin 3-*O*-glucoside in controlled aerobic batch conditions (Levisson et al., 2018). Except *E. coli* and *S. cerevisiae*, several other microorganisms have also demonstrated the ability to synthesize anthocyanins. Zha et al. (2018) constructed a recombinant *C. glutamicum* strain to produce cyanidin 3-*O*-glucoside from catechin by overexpression of ANS from *P. hybrida* and 3GT from *A. thaliana*. Further adjustment of the expression level of ANS and 3GT, process optimization, and improvement of UDP-glucose availability enabled the production of 40 mg/L cyanidin 3-*O*-glucoside (Zha et al., 2018). In another study, the *Lactococcus lactis* was engineered to produce some unusual anthocyanins using tea as substrate. Various red–purple cyanidin and delphinidin, orange, and yellow pyranoanthocyanidins were produced at a total titer of 1.5 mg/L by the end of 16 h (Solopova et al., 2019).

## Discussion and Perspectives

For the past few years, with the increasing knowledge about the natural biosynthetic pathways of flavonoids and the rapid development of synthetic biology and metabolic engineering, more and more microbial cell factories have been constructed to produce flavonoids from their direct precursors or from renewable carbon sources (Zhu et al., 2014; Wu et al., 2016b). However, commercial and industrial scale-up synthesis of those chemicals has yet been achieved because of the low product titer, yield, and productivity. In order to greatly improve the flavonoids titer in microorganisms, several challenges need to be addressed. First, the enzymes involved in flavonoids biosynthetic pathway, especially cytochrome P450s and postmodification enzymes, are usually redox partner dependence and have lower enzyme activity and poor selectivity (Stahlhut et al., 2015; Delmulle et al., 2018), which greatly limited the production efficiency of flavonoids. Recently, a lot of strategies have been made to improve their catalytic efficiency and specificity, such as rational protein engineering (Li et al., 2019), directed evolution (Pandey et al., 2016), substrate engineering (Li et al., 2020), and redox-partner engineering (Stahlhut et al., 2015). However, those efforts showed little effects on tackling the problem. We believe that utilization of the promiscuous of some efficient bacterial non-P450 oxygenases and hydroxylases to replace the plant cytochrome P450 enzymes serves as a promising alternative. Second, the flavonoids biosynthetic pathway involves lengthy reaction steps and complicated regulations that significantly limit production yield. To overcome this obstacle, some researchers attempted to construct coculture or polyculture system to distribute metabolic burden by dividing the entire biosynthetic pathway into several independent host strains (Ganesan et al., 2017; Thuan et al., 2018; Wang et al., 2020). However, the metabolite transport limitation and the difficulty in keeping stable and reliable coculture system are the primary challenges for a wide-range application of this approach (Jones et al., 2016b, 2017). Therefore, it is quite important for further optimization of the current coculture systems or seeking novel strategies to overcome this issue. Third, the production efficiency of flavonoids biosynthetic pathway is also limited by the insufficient supply of the precursors, especially the aromatic amino acids and malonyl-CoA (Koopman et al., 2012; Delmulle et al., 2018). However, those precursors also serve as essential intermediates to support cell growth; thus, it is very important to precisely distribute the carbon flux between the central metabolic pathways and the heterologous biosynthetic pathways. Recently, construction of dynamic regulatory elements to fine-tune the carbon flux between cell growth and products synthesis has been shown as a powerful tool to improve microbial synthesis efficiency (Wu et al., 2014, 2016a; Zhou et al., 2019). In addition, oleaginous yeast strains including *Yarrowia*, *Candida*, *Lipomyces*, and *Rhodotorula* are able to accumulate a high level of malonyl-CoA (Lv et al., 2019b), which serve as the promising hosts for flavonoid biosynthesis. The rapid and continued development of advanced tools and technologies is expected to create new and exciting opportunities in achieving efficient and economical production of flavonoids.

## Author Contributions

HS and JW drafted the manuscript. XSh, JW, and XSu revised the manuscript. YY and QY supervised this work. All authors contributed to the article and approved the submitted version.

## Conflict of Interest

The authors declare that the research was conducted in the absence of any commercial or financial relationships that could be construed as a potential conflict of interest.
